# Botanical Adjuvants in Oncology: A Review on Natural Compounds in Synergy with Conventional Therapies as Next-Generation Enhancers of Breast Cancer Treatment

**DOI:** 10.3390/cimb48020170

**Published:** 2026-02-02

**Authors:** Hidaya Mansouri, Ahmed Irchad, Clarence Rubaka, Lydia Kisula, Abdou Azali Hamza, Elingarami Sauli

**Affiliations:** 1 The Nelson Mandela African Institution of Science and Technology (NM-AIST), Arusha P.O. Box 447, Tanzania; 2 National Research Institute for Agriculture, Fisheries and Environment (INRAPE), Ex CEFADER, M’dé, Ngazidja, Moroni P.O. Box 1406, Comoros; 3 Department of Chemistry, St. John’s University of Tanzania, Dodoma P.O. Box 47, Tanzania

**Keywords:** phytotherapy, natural compounds, breast cancer, conventional therapy, anticancer agents, nanocarrier-based delivery systems

## Abstract

Breast cancer remains a major global health challenge despite advances in chemotherapy, endocrine therapy, targeted therapy, and radiotherapy, which are frequently constrained by therapeutic resistance, cumulative toxicity, and high costs. Accumulating preclinical and translational evidence demonstrates that plant-derived natural compounds can synergistically enhance the efficacy of conventional treatments, improve tumor response, and potentially reduce adverse effects. This review critically synthesizes in vitro, in vivo, and emerging clinical studies from 2015 to 2025, focusing on key phytochemicals, including curcumin, epigallocatechin-3-gallate, resveratrol, kaempferol, genistein, and other bioactive molecules as stand alone agents and as mechanistically validated adjuvants to chemotherapy, hormonal therapy, and radiotherapy. These compounds exert complementary actions, including the inhibition of PI3K/Akt/mTOR and NF-κB signaling, induction of apoptosis and cell-cycle arrest, suppression of epithelial–mesenchymal transition, and modulation of drug resistance pathways. Preclinical studies consistently show that combination strategies enhance tumor inhibition and may permit cytotoxic dose reduction, mitigating systemic and cardiotoxic effects. Nanocarrier-based delivery systems further optimize solubility, bioavailability, and tumor targeting. Despite robust preclinical evidence, clinical translation is limited by variability in raw materials, lack of standardization, regulatory barriers, and scarce large-scale trials. This review emphasizes both the therapeutic promise and translational challenges of integrating natural compounds as synergistic adjuvants in evidence-based breast cancer therapy.

## 1. Introduction

Recent advances in oncology pointed out the potential of integrating natural compounds with conventional treatment modalities [1,2,3,4]. Breast cancer arises from uncontrolled proliferation of cells within the lobules or ducts of the breast, forming tumors that can metastasize through blood and lymphatic systems [5,6,7]. Although it affects both sexes, breast cancer is predominantly diagnosed in women and remains the most frequently diagnosed malignancy worldwide, with 2.3 million new cases and 670,000 deaths reported in 2022 [5,8,9].

Marked disparities exist between regions: high-income countries have achieved declines in mortality through improved detection and treatment, whereas low-income countries face rising incidence and disproportionately high mortality, reflecting limited access to healthcare resources [5,10,11]. Conventional therapies including chemotherapy, radiotherapy, and immunotherapy have demonstrated efficacy [5,6,11,12], yet are constrained by drug resistance, severe side effects, and reduced effectiveness in advanced disease [8,12,13,14,15].

Against this backdrop, natural compounds derived from plants are gaining attention as adjuncts to conventional therapies [10,16,17]. Historically, medicinal plants have served as invaluable sources of therapeutic agents [18,19,20], and phytochemicals such as polyphenols, flavonoids, terpenoids, and alkaloids have shown promise in modulating pathways central to cancer progression [19,20,21,22,23]. Their affordability and accessibility make them particularly relevant for low- and middle-income countries, where up to 80% of the population relies on plant-based materials for primary healthcare [18,19,20].

Nevertheless, the current literature reveals critical shortcomings: lack of standardized extraction protocols, variability in raw materials, and limited large-scale clinical trials hinder reproducibility and clinical translation. While evidence suggests synergistic interactions between natural compounds and conventional therapies, mechanistic understanding remains incomplete. Addressing these gaps is essential to optimize combination strategies. This review therefore explores the applications of natural compounds as therapy alone or in conjunction with chemotherapy, radiotherapy, and surgery, emphasizing mechanisms of action, translational challenges, and future directions to enhance breast cancer treatment efficacy.

## 2. Methodology of the Review

The literature search was focused on medicinal plants used to treat breast cancer. Comprehensive independent literature searches were conducted across various databases including Scopus, PubMed/Medline, Science Direct, and Google Scholar over the last 10 years (between 2015 and 2025). The selected search keywords were: breast cancer, anticancer plants, and traditional medicinal plant extracts for cancer, cancer conventional treatment, plants based used to treat cancer, breast carcinoma, tumor, malignancies, carcinoma, natural compounds, maceration, decoction, infusion, concoction, ethnobotanical survey in cancer cancerous, phytochemicals properties, anticancer bioactive compounds. These were used to search for published journal articles, books and international reports. Inclusion criteria for this review focused on peer-reviewed articles, clinical and preclinical research, as well as insightful discussions on molecular mechanisms and their clinical implications. To maintain the clarity and relevance of this work, exclusion criteria were directed to non-peer-reviewed articles and studies that did not align with our objectives. By extracting valuable data on molecular mechanisms, sources, preclinical and clinical evidence, we laid the groundwork for exploring potential synergies with conventional therapies, fostering a comprehensive understanding of this field.

## 3. Review

### 3.1. Natural Compounds: Classification and Sources

Natural compounds are chemical substances made by living organisms. When derived from plants, they are called phytochemicals, which are secondary metabolites with unique structures and important functions in ecological and biological processes [10,18,24,25,26,27]. Phytochemicals are typically classified into several categories, each with unique properties and potential health benefits for humans, including antioxidant, anti-inflammatory, and anticancer effects [4,24,28,29,30]. Phytochemical compounds are emerging as powerful allies in the fight against breast cancer, showcasing remarkable potential through a variety of mechanisms. Among these, flavonoids, alkaloids, terpenoids, saponins, lignans, and polyphenols stand out for their potent anticancer properties [4,17,28,29,31].

Flavonoids, for instance, are not just passive agents; they actively inhibit cancer cell proliferation and trigger apoptosis in malignant cells, making them a formidable force against tumor growth [32,33,34]. Alkaloids further elevate this arsenal by disrupting crucial signaling pathways integral to cancer progression [30,35,36,37]. Meanwhile, terpenoids leverage their anti-inflammatory and antioxidant effects to counteract the cancerous processes [38,39,40,41]. Saponins enhance the body immune response, potentially amplifying the effects of conventional therapies and providing a synergistic boost to treatment outcomes [24,42,43]. Lignans and polyphenols are equally impressive, adept at modulating essential cellular mechanisms such as cell cycle regulation and DNA repair, further establishing their role as key players in cancer therapy [44,45,46]. This diverse spectrum of bioactive compounds reinforces the urgent need to integrate plant-derived substances into breast cancer treatment strategies. Their multifaceted modes of action not only promise superior effectiveness but also offer the hope of reduced toxicity for patients. With ongoing research into these phytochemicals, we edge closer to developing innovative, more effective therapies that could revolutionize breast cancer treatment and improve patient outcomes. Sources of these beneficial compounds include a variety of leaves, fruits, roots, seeds, and bark, such as berries, citrus fruits, leafy greens, and cruciferous vegetables [24,30,47]. Spices like turmeric and ginger, along with herbs like parsley and oregano, are also rich in bioactive compounds [24,48,49]. Additionally, legumes and grains, particularly beans, lentils, oats, and flaxseeds, provide a wealth of phytochemicals that have been studied for their potential anticancer effects (Table 1).

Overall, the potential of phytochemicals and natural compounds in breast cancer treatment lies in their ability to target multiple pathways involved in cancer development. By incorporating a diet rich in fruits, vegetables, spices, herbs, legumes, grains, tea, wine, nuts, and seeds, individuals can harness the power of these natural compounds to support their health and potentially reduce the risk of breast cancer development.

### 3.2. From Bench to Bedside: Plant-Based Compounds in Breast Cancer Therapy

Natural products remain a cornerstone of oncology, with approximately 75% of anticancer agents originating from plant sources [60,61,62]. In breast cancer therapy, natural compounds have demonstrated significant potential, culminating in the regulatory approval of several plant-derived drugs [4,7,63,64]. These agents exemplify the therapeutic value of natural compounds in targeted cancer treatment, operating through distinct bioactive mechanisms that enhance efficacy and specificity [7,26,48,49,65,66,67]. Table 2 provides an overview of representative drugs, their botanical origins, mechanisms of action, clinical application stages, and ongoing trials in breast cancer management.

### 3.3. Mechanistic Insights, Preclinical Evidence, and Translational Perspectives of Plant-Derived Compounds in Breast Cancer Intervention

The exploration of natural compounds in both preclinical and clinical settings highlights a paradigm shift toward the integration of phytochemicals in breast cancer therapy [31,61,62,96,97]. Among these, curcumin the principal bioactive constituent of turmeric (*Curcuma longa*) has attracted considerable attention for its dual anti-inflammatory and antioxidant properties [83,84,85]. Evidence indicates that curcumin not only suppresses breast cancer cell proliferation but also induces apoptosis, supporting its potential as an adjunct to conventional treatment regimens [83,84]. In vitro studies consistently demonstrated that curcumin modulates multiple oncogenic signaling pathways, including inhibition of PI3K/Akt/mTOR and NF-κB activity, cell-cycle arrest at the G2/M phase via regulation of CDC25 and p21, and suppression of metastatic processes through downregulation of matrix metalloproteinases (MMPs) and VEGF [98,99,100,101,102]. These effects have been observed across diverse breast cancer subtypes, including ER-positive (MCF-7), HER2-positive, and triple-negative (MDA-MB-231) cell lines [98,99,100]. In vivo investigations further validate its efficacy, showing that nanoparticle-based formulations of curcumin reduce tumor volume and inhibit growth in experimental models [103,104,105]. A major translational challenge remains its poor solubility and bioavailability, which advanced nano-delivery systems aim to address. Although clinical evidence is less extensive than preclinical data, early trials suggest that curcumin particularly in nanoformulations or in combination with chemotherapeutics such as docetaxel can delay disease progression, lower tumor markers, and alleviate treatment-related side effects, including radiation dermatitis [83,84,85]. Therefore, these findings position curcumin as a promising multi-target agent, bridging mechanistic insights from cellular studies with encouraging outcomes in early clinical investigations, and warranting further rigorous evaluation to define its standardized role in breast cancer management.

Epigallocatechin gallate (EGCG), the predominant polyphenol in green tea, exhibits broad anticancer activity in breast cancer, supported by in vitro, in vivo, and clinical evidence. Preclinical studies consistently demonstrated its anti-proliferative, pro-apoptotic, and anti-metastatic effects across multiple breast cancer subtypes [106,107,108]. These actions are mediated through modulation of key oncogenic pathways, including inhibition of PI3K/Akt, NF-κB, MAPK, and EGFR signaling, as well as downregulation of β-catenin in triple-negative breast cancer (TNBC) cells such as MDA-MB-231 [106,109]. EGCG also enhances the cytotoxicity of conventional chemotherapeutics, underscoring its synergistic potential. In vivo xenograft models corroborate these findings, showing significant suppression of tumor growth and metastasis [71,106,109]. Translational challenges remain, particularly EGCG’s poor oral bioavailability and rapid systemic metabolism, which constrain its therapeutic application. Clinically, epidemiological studies suggest an association between green tea intake and reduced breast cancer risk, though direct antitumor efficacy trials are limited [109]. The strongest clinical evidence to date derives from supportive care studies, where topical EGCG formulations have proven safe and effective in reducing radiation-induced dermatitis among breast cancer patients receiving radiotherapy [110,111]. Collectively, these findings position EGCG as a promising multi-target agent with well-defined preclinical mechanisms and emerging clinical utility, while highlighting the need for innovative pharmacokinetic strategies to enable its development as a systemic therapy.

Paclitaxel, a diterpenoid originally isolated from the Pacific yew tree (*Taxus brevifolia*), remains distinct among natural compounds due to its long-established role as a first-line chemotherapeutic agent in breast cancer [68,112]. Its mechanism of action is well characterized: paclitaxel binds to β-tubulin, promoting microtubule polymerization and stabilization, which induces cell-cycle arrest at the G2/M phase and triggers apoptosis [68,112,113]. This activity has been extensively validated in vitro across diverse breast cancer cell lines, including luminal (MCF-7), HER2-positive (SKBR-3), and triple-negative (MDA-MB-231) models [68,112,113,114,115]. Beyond its cytotoxic effects, in vitro studies highlight its capacity to inhibit migration and invasion, as well as its synergistic potential when combined with targeted agents such as miRNA inhibitors in advanced delivery systems. In vivo investigations confirmed that paclitaxel and its novel formulations effectively suppress tumor growth and metastasis in animal models [68,112,113,114,115,116,117]. Clinically, paclitaxel has been a cornerstone of breast cancer therapy since the 1990s, with proven efficacy across neoadjuvant, adjuvant, and metastatic settings. Ongoing trials continue to refine its application, aiming to enhance progression-free survival and patient outcomes. Nevertheless, resistance mechanisms including overexpression of drug efflux pumps (e.g., P-glycoprotein) and tubulin mutations pose significant challenges, driving intensive research into innovative formulations and combination strategies to overcome these limitations [116,118,119,120].

Quercetin, a dietary flavonoid abundant in vegetables and fruits, is recognized for its anti-inflammatory and antioxidant properties [121,122]. Preclinical evidence highlights its potential to enhance the efficacy of conventional chemotherapies while mitigating treatment-related side effects, positioning it as a promising candidate in breast cancer therapy [24,112,122,123]. In vitro studies demonstrate that quercetin exerts multi-targeted anticancer effects by inducing apoptosis, promoting cell-cycle arrest, and inhibiting invasion across diverse breast cancer cell lines, including MCF-7, MDA-MB-231, and SK-BR-3. These effects are mediated through modulation of critical signaling pathways such as PI3K/Akt, Wnt/β-catenin, and MAPK/ERK [121,122,123,124]. In vivo xenograft models further corroborate its activity, showing reductions in tumor volume and metastatic spread. Although robust clinical trials of quercetin monotherapy remain limited, ongoing investigations such as a Phase II trial (NCT06355037) assessing its combination with chemotherapy in metastatic triple-negative breast cancer (mTNBC) highlight its translational potential [121,123,124].

Camptothecin, a naturally occurring alkaloid, and its analogs have emerged as potent candidates in breast cancer therapy. Their anticancer activity is primarily mediated through inhibition of DNA topoisomerase I, resulting in replication-associated DNA damage and apoptosis [125]. This mechanism has been validated in vitro across major breast cancer subtypes, including ER-positive (MCF-7), HER2-positive (SK-BR-3), and triple-negative (MDA-MB-231) cell lines [126]. In vivo studies further demonstrated that advanced nano-delivery systems, such as polymeric micelles and liposomes, enhance tumor selectivity, improve solubility, and achieve superior suppression of tumor growth and metastasis in xenograft models like 4T1 and MDA-MB-231 [125,126,127,128,129]. Jointly, these findings highlight camptothecin’s translational promise, emphasizing the need for continued development of optimized formulations to fully realize its therapeutic potential in breast cancer management.

Beta-carotene, a carotenoid widely present in fruits and vegetables, has shown promising anticancer potential in breast cancer research. In vitro studies demonstrate its cytotoxic activity across ER-positive (MCF-7), HER2-positive (SK-BR-3), and triple-negative (MDA-MB-231) cell lines, characterized by apoptosis induction, cell-cycle arrest, and metabolic modulation [130,131,132,133]. In vivo investigations, particularly those employing carotenoid mixtures containing beta-carotene derivatives, reveal tumor growth suppression and regulation of oncogenic pathways such as HER2 and p53 in rat models of HER2-positive breast carcinoma [130,131,132,133]. Complementary epidemiological evidence consistently associated the elevated dietary intake and circulating levels of beta-carotene with reduced breast cancer risk. Taken together, these findings support beta-carotene as a compound with mechanistically defined antitumor properties and a strong chemopreventive association, meriting the further exploration of its translational and therapeutic potential [130,131,132,133].

Sulforaphane, a bioactive isothiocyanate abundant in cruciferous vegetables such as broccoli, is under active investigation in both preclinical and clinical settings for its anticancer and antioxidant properties [134,135,136,137,138,139]. Evidence indicates that sulforaphane can inhibit cancer cell growth and mitigate oxidative stress, positioning it as a promising candidate in breast cancer therapy [134,135,136,137,138,139]. A recent systematic review encompassing 12 in vitro studies, 5 in vivo animal models, and 3 randomized controlled trials (RCTs), confirmed its ability to induce apoptosis, promote cell-cycle arrest, and suppress metastasis, particularly in stem-like triple-negative breast cancer (TNBC) cells [140]. Although the clinical trials were limited in scale, they supported sulforaphane’s safety profile.

Silibinin, the principal active constituent of milk thistle (*Silybum marianum*), has been extensively investigated for its therapeutic potential in oncology and hepatology [141]. Preclinical studies revealed that silibinin exerts anticancer effects by modulating multiple signaling pathways, including PI3K/Akt, STAT3, and Wnt/β-catenin, resulting in apoptosis induction, cell-cycle arrest, reduced angiogenesis, and suppression of inflammatory responses [141,142]. It also enhances drug sensitivity through immune modulation, supporting its role as a multi-target agent. Translational relevance is highlighted by ongoing clinical research, such as the SILMET trial (NCT05689619), which is assessing silibinin in breast cancer patients with brain metastases to prevent recurrence via STAT3 inhibition. In addition to its anticancer activity, silibinin’s hepatoprotective, anti-inflammatory, and antioxidant properties reinforce its broader clinical utility [141,142,143]. Overall, silibinin exemplifies the expanding role of phytochemicals in modern medicine, bridging traditional applications with evidence-based validation in preclinical and clinical studies.

### 3.4. Molecular Pathways Unveiled Through Natural Compounds

**Figure 1:**
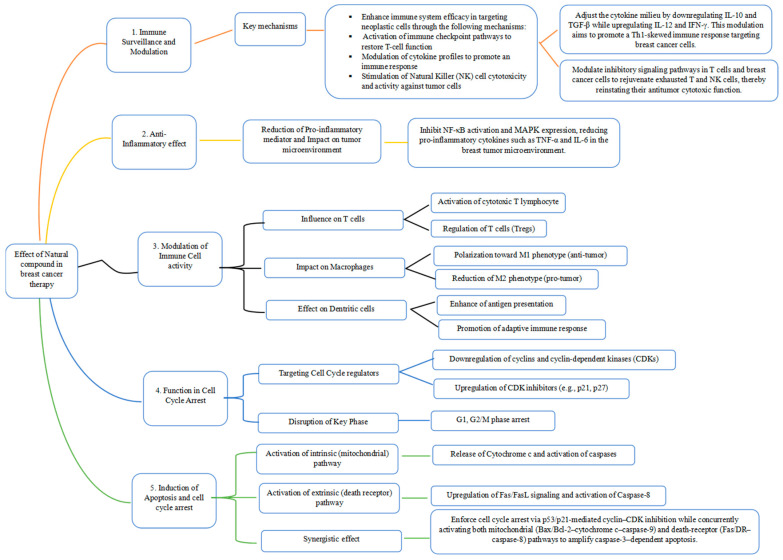
Mechanistic path for natural compounds in Breast Cancer therapy.

Plant-derived natural compounds are increasingly recognized as powerful agents in both the treatment and prevention of cancer. Their diverse bioactive properties greatly influence tumor-associated cellular pathways, positioning them as vital candidates for innovative therapeutic strategies and effective cancer prevention [18,21,24,47,48,49,66]. These phytochemicals actively modulate key signaling cascades, suppress cancer cell proliferation, induce apoptosis, and enhance antitumor immune responses [17,29,144]. A clear understanding of these mechanisms, as well as their potential synergistic interactions, is essential to fully harness the therapeutic promise of natural compounds in clinical applications. The principal molecular pathways through which these agents exert their effects are summarized in Figure 1.

#### 3.4.1. Immune Surveillance and Modulation

Phytocompounds exhibit considerable potential in modulating the functional activity of diverse immune cell subsets, thereby augmenting the immune system’s capacity to recognize and eradicate neoplastic cells more effectively [4,29,31,96,97,145]. Curcumin and resveratrol have gained significant attention for their capacity to enhance the functionality of natural killer (NK) cells, which play a pivotal role in immune surveillance and eradication of tumors, particularly in breast cancer [83,84,85,104]. Their modulation of NK cell activity underscores their potential in immunotherapeutic strategies aimed at improving anti-tumor responses [83,84,85,87,88,89]. Controlled co-culture experiments utilizing human NK-92 cells, an established model for investigating natural killer cell functionality, alongside MDA-MB-231 breast carcinoma cells, revealed that curcumin enhances the prevalence of CD16^+^ CD56^−^ NK cells [83,84,85,87,88,89]. This subset of NK cells is associated with increased cytotoxic potential, indicating curcumin’s promising role in modulating NK cell activity against cancer cells [83,84,85,87,88,89]. This enhanced functionality of NK cells is primarily attributed to the activation of specific intracellular signaling pathways, including STAT4 and STAT5. These pathways play an integral role in promoting NK cell proliferation, differentiation, and cytotoxicity.

Curcumin has shown to downregulate the phosphorylation of extracellular signal-regulated kinase (pERK) and phosphoinositide 3-kinase (PI3K) within tumor cells, potentially diminishing survival signals and increasing their susceptibility to immune-mediated destruction [83,84,85]. This dual mechanism not only enhances the effectiveness of natural killer (NK) cells in targeting cancer cells but also disrupts the survival mechanisms of the tumor cells themselves. Furthermore, a recent study by Ding et al. (2025) highlights the significant role of resveratrol in enhancing the cytotoxic activity of NK cells against breast cancer [146]. The research indicates that resveratrol effectively downregulates microRNA miR-17-5p, leading to increased expression of the NKG2D ligand ULBP2 on the surface of tumor cells. This crucial mechanism activates the MINK1/JNK/c-Jun signaling pathway, significantly increasing the vulnerability of cancer cells to destruction by NK cells. Resveratrol emerges as a promising agent for enhancing tumor clearance, potentially improving outcomes in breast cancer patients. Other phytochemicals, including quercetin, luteolin, and curcumin, also effectively stimulate T cell responses, aiding in the recognition and elimination of tumor cells [84,85,122,123,147]

Furthermore, epigallocatechin gallate (EGCG), a potent green tea polyphenol, has demonstrated significant ability to enhance CD4^+^ and CD8^+^ T cell activation and proliferation [106,107,108,111]. This enhancement is crucial for optimizing immunotherapy, which relies on robust T cell responses against malignancies [107,108]. Elevated T cell expression is consistently associated with better clinical outcomes, highlighting the importance of boosting T cell functionality in cancer treatment strategies.

On the other side, phytocompounds significantly contribute to the enhancement of dendritic cells’ maturation and functionality, which are essential for effective antigen presentation and the activation of T-cells. This intricate process is important for the establishment of a vigorous and adaptive anti-tumor immune response [24,33,51,148]. Dendritic cells serve as the initiators of de novo T-cell immunity and are responsible for priming antigen-specific T-cells, thereby laying the foundation for a targeted immune attack against tumors. In addition, dendritic cells have been recognized as pivotal regulators that shape the body’s response to immune checkpoint blockade therapies and a variety of cancer immunotherapies, highlighting their importance in advancing cancer treatment strategies.

#### 3.4.2. Anti-Inflammatory Effects

Chronic inflammation is a major contributor to cancer initiation, progression, and metastasis, creating a tumor-supportive microenvironment (TME) [54,55,149]. The anti-inflammatory properties of phytocompounds are supported by extensive in vitro signaling studies, in vivo inflammatory tumor models, and limited clinical biomarker analyses [54,55,150].

Curcumin and quercetin inhibit key inflammatory signaling pathways, including nuclear factor-κB (NF-κB) and mitogen-activated protein kinase (MAPK), as demonstrated in cell-based assays using breast cancer and immune cell lines [83,84,85,104,121,123,124]. These effects translate into reduced production of pro-inflammatory cytokines and chemokines that drive tumor growth and metastasis. In vivo studies further confirm that suppression of these pathways is associated with reduced tumor-associated inflammation and delayed tumor progression [83,124].

In addition to anti-inflammatory activity, these compounds exhibit potent antioxidant effects. In vitro redox assays and animal models show effective scavenging of reactive oxygen species (ROS), resulting in reduced oxidative stress, DNA damage, and inflammation hallmarks of neoplastic tissues [100,151]. Clinical studies examining dietary flavonoid intake have reported improvements in systemic oxidative stress markers, supporting their relevance in cancer prevention and supportive care [151].

Salvianolic acid B (Sal-B), derived from Salvia miltiorrhiza (Danshen), exhibits anti-inflammatory and antitumor activity across multiple experimental systems [152]. In vitro studies demonstrate suppression of oxidative stress and inflammatory mediators, including tumor necrosis factor-α (TNF-α) and matrix metalloproteinase-8 (MMP-8) [153]. These findings are corroborated by in vivo tumor models, which show reduced tumor growth and increased apoptosis via upregulation of caspase-3 and p53 [152,153]. Although clinical data remain limited, Sal-B has shown anti-inflammatory benefits in clinical settings, supporting further oncological investigation.

#### 3.4.3. Modulation of Immune Cell Activity and Function of Natural Compounds in Cell Cycle Arrest for Cancer Management

Natural compounds directly regulate the activity of key immune cell subsets involved in antitumor defense. EGCG, quercetin, and curcumin enhance the activation and cytotoxic function of CD8^+^ cytotoxic T lymphocytes in breast cancer models, promoting tumor cell killing [83,85,98,106,107,123,124]. Alongside, curcumin, resveratrol, genistein, and EGCG reduce the number and suppressive activity of regulatory T cells (Tregs) and myeloid-derived suppressor cells (MDSCs), thereby alleviating immunosuppression within the TME [83,85,98,106,107,123,124].

Macrophage polarization is another critical immunomodulatory target. Polyphenols such as resveratrol, EGCG, quercetin, and curcumin promote polarization toward the M1 (antitumor) phenotype while suppressing M2 (protumor) macrophages in murine breast cancer models. This phenotypic shift enhances antigen presentation, nitric oxide production, and inflammatory cytokine release conducive to tumor rejection [89,154].

Furthermore, natural compounds enhance dendritic cell maturation and antigen-presenting capacity. Curcumin and EGCG increase expression of co-stimulatory molecules and improve antigen presentation, leading to more effective priming of adaptive immune responses and strengthening immune memory against breast cancer antigens [24,155,156,157].

#### 3.4.4. Induction of Apoptosis and Cell Cycle Arrest

Apoptosis, or programmed cell death, is a fundamental biological process responsible for maintaining cellular homeostasis and eliminating damaged or potentially malignant cells, thereby preventing oncogenic transformation and tumor progression [5,158]. This highly regulated mechanism ensures controlled cellular turnover and tissue integrity through the activation of specific intracellular signaling pathways [2,159,160]. A growing body of evidence demonstrates that phytocompounds effectively induce apoptosis in breast cancer cells by engaging both intrinsic (mitochondrial) and extrinsic (death receptor–mediated) apoptotic pathways [159].

Epigallocatechin gallate (EGCG) is among the most studied phytochemicals with anticancer activity. In vitro studies using breast cancer cell lines, including MCF-7 and MDA-MB-231, showed that EGCG induces mitochondrial-mediated apoptosis by upregulating pro-apoptotic proteins (Bax, p53, and caspase-3) while suppressing anti-apoptotic factors such as Bcl-2 and cIAP2 [106,107]. These molecular effects result in caspase activation and apoptotic cell death. In vivo studies further corroborate these findings, demonstrating that EGCG reduces tumor growth and enhances apoptotic indices in breast cancer xenograft models [106,107,108]. Limited clinical studies and epidemiological data also suggest an inverse association between green tea consumption and breast cancer progression, although definitive clinical evidence remains limited.

In addition to apoptosis induction, natural compounds inhibit breast cancer cell proliferation by enforcing cell-cycle arrest [55]. Compounds such as curcumin, quercetin, EGCG, berberine, resveratrol and genistein have been shown in vitro to modulate cyclins, cyclin-dependent kinases (CDKs), and CDK inhibitors, thereby disrupting cell-cycle progression [87,89,106,107,154,160,161,162,163,164]. Genistein, in particular, induces G2/M phase arrest in breast cancer cells through CDK inhibition and p21 upregulation, effects that have been validated in murine tumor models and are associated with reduced tumor burden and enhanced apoptotic signaling [163,165].

Low-molecular-weight phenolic acids, including caffeic, gallic, ferulic, p-coumaric, chlorogenic, and sinapic acids, also exhibit significant anti-breast cancer activity [56,57]. In vitro studies demonstrated that caffeic acid induces apoptosis and G1/S phase arrest via reactive oxygen species generation and mitochondrial membrane depolarization in both hormone-dependent and triple-negative breast cancer cell lines [166,167]. These effects are further supported by in vivo studies showing reduced tumor growth and metastatic potential, particularly when caffeic acid is combined with paclitaxel [68,112,114]. Similarly, gallic acid promotes caspase-dependent apoptosis, suppresses cyclin D1 expression, and inhibits angiogenesis through downregulation of VEGF signaling in xenograft models of triple-negative breast cancer [168]. Ferulic acid, alone or in combination with p-coumaric acid, suppresses tumor invasion and metastasis by inhibiting MMP-9 expression and disrupting the PI3K/Akt/NF-κB signaling pathway, with consistent evidence from orthotopic mouse models [169].

Emerging evidence also indicates that chlorogenic and sinapic acids modulate epigenetic mechanisms by inhibiting histone deacetylases, thereby enhancing chemosensitivity and targeting breast cancer stem cell self-renewal pathways in preclinical models [60,170]. While clinical data remain scarce, these findings highlight their potential as adjuvant agents in integrative breast cancer therapy. Despite these encouraging findings, further mechanistic studies and well-designed clinical trials are required to fully elucidate the molecular targets, assess long-term safety, and define optimal dosing regimens and combination strategies. Such investigations are essential for translating the anticancer potential of these natural compounds into clinically effective interventions that improve outcomes in breast cancer management.

#### 3.4.5. Synergistic Effects of Natural Compounds with Conventional Therapies

One of the most promising attributes of natural compounds is their potential to enhance the efficacy of conventional breast cancer therapies, including chemotherapy, hormonal therapy, and radiotherapy [7,23,47,117,122]. Conventional treatment modalities have substantially improved survival outcomes; however, they are frequently associated with treatment-related toxicity and the development of therapeutic resistance. Chemotherapy involves the use of cytotoxic agents such as cyclophosphamide and doxorubicin to eliminate rapidly dividing cancer cells [3]. Hormonal therapy employs endocrine-blocking agents, including tamoxifen, raloxifene, and aromatase inhibitors such as anastrozole, to suppress estrogen-driven proliferation in hormone receptor–positive tumors and reduce recurrence risk [158,171]. Radiotherapy utilizes high-energy ionizing radiation to induce irreparable DNA damage in cancer cells and is sometimes combined with systemic therapies, including capecitabine, in selected high-risk clinical settings [158,171].

Despite their therapeutic benefits, conventional treatments may lead to cumulative adverse effects, including cardiotoxicity, particularly in patients receiving anthracyclines, HER2-targeted therapies, or chest irradiation [3,172,173]. These toxicities can significantly compromise long-term quality of life [6,7]. In addition, a substantial proportion of patients with estrogen receptor–positive breast cancer reported in some studies to be up to 30–40% develop resistance to tamoxifen or aromatase inhibitors within five years, underscoring the urgent need for improved therapeutic strategies [3,158]. Given these circumstances, it is imperative to reevaluate the strategies and consider alternative therapies that could potentially enhance patient outcomes and reduce toxicity.

Although numerous natural compounds have been embraced as viable treatments within the realm of oncology, the world of potential drug candidates is vast, encompassing thousands of compounds that have not yet undergone official clinical trials for a variety of reasons. This sheer volume of natural substances makes it impossible to create an exhaustive inventory within the limits of this review. Consequently, Table 3 spotlight selected examples, highlighting recent discoveries of compounds that have demonstrated promising effects against breast cancer. A diverse array of compounds has emerged as crucial players in the suppression of cancer-activating pathways, inhibiting oncogenes that contribute to tumor formation, and activating apoptotic pathways within malignant cells.

## 4. Advancing Breast Cancer Therapy Through Nanoformulated Natural Compounds

Nanoformulation of plant-derived compounds addresses key pharmacokinetic and pharmacodynamic limitations poor aqueous solubility, rapid metabolism, short plasma half-life, and off-target distribution thereby enabling these agents to act as effective adjuvants to conventional breast cancer therapies [184,185]. Common nanoparticles include liposomes, polymeric nanoparticles, solid lipid nanoparticles (SLN) and nanostructured lipid carriers (NLC), polymeric micelles, dendrimers, metallic nanoparticles (gold, iron oxide), metal–organic frameworks, and biologically derived vesicles (exosomes) [184,186]. Each nanoparticle affords specific advantages: liposomes and SLNs improve solubility and biocompatibility; polymeric nanoparticles particles enable controlled release and tunable degradation; micelles solubilize highly hydrophobic phytochemicals; and inorganic nanoparticles support imaging and theranostic applications [184,186].

Mechanistically, the therapeutic performance of nanoformulated natural compounds in breast cancer is strongly influenced by the route of administration, which governs biodistribution, tumor accumulation, and systemic toxicity. Intravenous (IV) delivery remains the most widely investigated route, as it enables direct systemic circulation, exploitation of the enhanced permeability and retention effect, and efficient tumor targeting in orthotopic and xenograft models [187,188]. IV-administered liposomal or polymeric nanoparticles encapsulating curcumin, epigallocatechin-3-gallate, or resveratrol have demonstrated prolonged circulation time, improved tumor uptake, and superior synergy with chemotherapeutic agents such as doxorubicin and paclitaxel, while reducing cardiotoxicity and off-target exposure [179,189]. Oral delivery, although challenged by gastrointestinal degradation and first-pass metabolism, has shown promise through lipid-based nanoparticles, nanoemulsions, and polymeric micelles that enhance intestinal absorption and bioavailability of hydrophobic phytochemicals, supporting long-term adjuvant therapy [187,190]. Therefore, optimization of administration routes is essential to maximize the therapeutic synergy of nanoformulated natural compounds with conventional breast cancer treatments and to facilitate clinical translation.

## 5. Challenges and Future Direction for Natural Compounds in Breast Cancer Therapy

The clinical translation of botanical agents in breast cancer is primarily constrained by raw-material variability and the lack of standardized extraction and formulation processes [1,26,63,65,191,192]. Phytochemical profiles vary substantially with geographic origin, cultivar, growth conditions, harvest timing, and post-harvest handling; such heterogeneity undermines reproducibility between batches and across laboratories. Where studies rely on crude extracts, the complex composition can obscure the contribution of individual bioactive molecules, complicating dose optimization, toxicity assessment, and mechanistic interpretation [24,48,49,62]. To address these issues, standardized sourcing practices, validated chemical fingerprints (e.g., HPLC, LC-MS, or GC-MS), quantitative marker-compound assays, and batch-release criteria are essential to ensure consistency and reliable bioactivity across experiments [193,194,195].

Translational gaps between in vitro, in vivo, and clinical studies further limit the predictability of laboratory findings in humans. In vitro studies often employ concentrations and exposure times that are pharmacologically unattainable in patients, while in vivo models may not fully recapitulate human tumor biology, metabolism, or tumor host interactions [125,196]. Clinical trials, by contrast, must account for inter-patient heterogeneity, comorbidities, drug–drug interactions, and long-term safety, factors frequently absent in preclinical studies [31,114]. To bridge these gaps, integrative study designs incorporating physiologically relevant dosing, pharmacokinetic/pharmacodynamic (PK/PD) modelling, validated biomarkers of target engagement, and robust preclinical models (orthotopic xenografts, patient-derived xenografts) are recommended.

Pharmacoeconomic and regulatory considerations are additional barriers to the adoption of natural compounds [62,197]. Although plant material may be inexpensive, the costs associated with standardized cultivation, extraction, quality control, formulation, toxicology, and late-phase clinical trials can be substantial. Limited patentability may further reduce private investment, emphasizing the need for early cost-effectiveness analyses and strategies demonstrating improvements in clinical outcomes, such as reduced chemotherapy dosing, decreased toxicity, or enhanced quality-adjusted life years. Innovative formulation approaches, including liposomal, polymeric, and lipid-based nanoparticles, offer promising solutions to improve solubility, bioavailability, circulation time, and tumor-specific accumulation. Combined with high-throughput screening, in silico modeling, and AI-driven network pharmacology, these strategies can accelerate the identification of synergistic combinations and predictive biomarkers. Multidisciplinary collaboration among botanists, pharmacologists, formulation scientists, clinicians, and health economists will be critical to designing rigorous, biomarker-guided clinical trials, ultimately enabling natural compounds to complement conventional breast cancer therapies while ensuring reproducibility, safety, and economic feasibility.

## 6. Conclusions

The drug discovery landscape is evolving to become more competitive, integrating traditional ethnopharmacology with innovative synthetic methods. This review supports three principal conclusions. First, substantial in vitro and in vivo evidence demonstrates that selected natural compounds can synergistically enhance the efficacy of conventional breast cancer therapies by modulating apoptosis, cell-cycle regulation, oncogenic signaling pathways, and drug resistance mechanisms. Second, nano-delivery systems markedly improve the bioavailability, tumor targeting, and therapeutic index of phytochemicals, thereby strengthening their clinical relevance as adjuvant agents. Third, clinical translation remains limited by variability in raw materials, the lack of standardization, and insufficient large-scale, biomarker-guided clinical trials. Based on these findings, several practical recommendations emerge. Priority should be given to clinical evaluation of well-characterized compound classes, particularly polyphenols (e.g., curcumin, epigallocatechin-3-gallate, resveratrol), flavonoids (e.g., quercetin, kaempferol), and isoflavones (e.g., genistein), administered in standardized or nanoformulated forms. The combination of regimens pairing these agents with established chemotherapeutics (doxorubicin, paclitaxel), endocrine therapies (tamoxifen, aromatase inhibitors), or radiotherapy warrant focused investigation. Future clinical trials should adopt rigorous quality control, pharmacokinetic profiling, and biomarker-driven designs to validate efficacy, safety, and cost-effectiveness.

## Figures and Tables

**Table 1:** Diversity of natural compounds studied for breast cancer prevention.

Type of Compound	Nature of Compound	Subclass of Compound	Example Compounds	Example of Plants	Specific Cell Lines & Subtypes Studied	Mechanism of Action	References
Flavonoids	Class of polyphenolic secondary metabolites found in plants, and commonly consumed in the diets of humans. The general structure of flavonoids is a fifteen-carbon skeleton, containing two benzene rings connected by a three-carbon linking chain. Therefore, they are depicted as C6-C3-C6 compounds.	Flavones, Flavonols, Flavanones, Isoflavones, Anthocyanidins	Quercetin, Kaempferol, Luteolin, Apigenin, Genistein, Epigallocatechin gallate (EGCG), Silibinin, Biochanin A, Icaritin, Hesperetin	Commonly found in fruits, vegetables, teas, and herbs such as Green tea, Citrus fruits, Cereals, and legumes	MCF-7 (ER/PR +), MDA-MB-231 (TNBC), T-47D, SK-BR-3 (HER2+)	Induce apoptosis by activating intrinsic mitochondrial cascades and inhibit cell proliferation through suppression of PI3K/Akt and MAPK pathways. block angiogenesis and metastasis through downregulation pathway, offering chemopreventive effects and acting adjunctively with chemotherapy in both early- and late-stage treatment.	[24,25,32,33,50]
Alkaloids	Lager cluster of organic secondary metabolites that contain a nitrogen atom and have diverse and important pharmaceutical effect.	Piperidine, Quinoline, Indole, Isoquinoline, Tropan	Vincristine, Vinblastine, Camptothecin, Topotecan, Irinotecan, Berberine, Noscapine, Oxymatrine, Artemisinin	Primarily plant-derived like *Madagascar periwinkle* *Black pepper* (*Piper nigrum*), *long pepper* (*Piper longum*)	MDA-MB-231 (TNBC), MCF-7 (ER/PR+), MCF-10A (normal)	Synergistically inhibit STAT3 phosphorylation & induce apoptosis. Activate caspase-8/caspase-9, modulate Bcl-2/Bax. Inhibit microtubule polymerization	[36,37,51,52,53]
Terpenoids	Diverse, lipophilic compounds derived from isoprene units; variable structures	Monoterpenoids, Sesquiterpenoids, Diterpenoids, Tetraterpenoids	Andrographolide, Paeoniflorin, Tanshinone IIA, Celastrol, Lycopene	Widely distributed among plants (and some marine organisms) *Pacific yew* (source of paclitaxel), Algae, mushrooms and lichens	MCF-7 (ER/PR+), MDA-MB-231 (TNBC), MDA-MB-468 (TNBC), 4T1 (murine TNBC)	Inhibit PI3K/Akt/mTOR pathway (e.g., paeoniflorin, Tanshinone IIA). Modulate MAPK/ERK, Bax/Bcl-2/caspase-3, NF-κB/EMT pathways. Induce autophagy (e.g., artemisinin, oridonin).	[38,39,40,41]
Saponins	Secondary metabolites that are heat-stable, amphiphilic. Glycosides comprising a sugar portion linked to a triterpene or steroid aglycone	Triterpenoid saponins, Steroid saponins	Saikosaponin-A, Dioscin, α-Hederin, Ginsenosides	Ginseng, Quinoa, and Fenugreek	MDA-MB-231 (TNBC), MCF-7 (ER/PR+)	Promote apoptosis, inhibit angiogenesis, modulate the immune system, and improve the efficacy of chemotherapeutic agents; evaluated in both clinical settings and preclinical models for synergistic effects.	[24,42,43]
Lignans	Polyphenolic substances with phytoestrogenic and antioxidant properties	Dibenzylbutane, Dibenzylbutyrolactone, Furanofuran	Secoisolariciresinol diglucoside (SDG), Enterolactone (ENL), Macelignan	Mainly sourced from high-fiber plants, seeds, and whole grains like Flaxseed and Sesame seeds	MDA-MB-231 (TNBC), MCF-7 (ER/PR+), T-47D (ER/PR+)	Modulate estrogen metabolism; induce cell cycle arrest and apoptosis; particularly beneficial in hormone-responsive breast cancer treatment across both preclinical and clinical phases	[44,45,46]
Polyphenols	Broad class featuring multiple phenol groups; includes both flavonoids and non-flavonoids with antioxidant and anti-inflammatory actions	Phenolic acids, Stilbenes, Tannins	Curcumin, Ellagic acid, Gallic acid, Resveratrol (also a stilbene), Chlorogenic acid, Caffeic acid, Ursolic acid,	Grapes, Berries, Green tea, Turmeric	MCF-7 (ER/PR+), MDA-MB-231 (TNBC), BT-474 (HER2+)	Target cellular signaling pathways to inhibit carcinogenesis, promote apoptosis, and reduce oxidative stress; deployed as chemopreventive agents and in adjunct therapy in both early and advanced stages.	[54,55,56,57,58,59]

**Table 2:** Plant-Derived Anticancer Drugs: Comprehensive Overview.

Drug (Generic)	Source Plant/Origin	Cancer Type	Mechanism of Action	Treatment Stage	Regulatory Organization	Ongoing Trials	References
Paclitaxel	Bark of *Taxus brevifolia* (Pacific yew)	Breast First-line for multiple subtypes (e.g., TNBC, HER2+)	Microtubule stabilization → mitotic arrest, apoptosis	Approved drug (first-line/metastatic Breast Cancer)	FDA, EMA	Nano-formulation strategies ongoing	[24,68]
Docetaxel	Needles of *Taxus baccata* (European yew)	Breast (First-line and metastatic breast cancer)	Microtubule stabilization → mitotic arrest	Approved drug	FDA, EMA	Combination chemotherapy regimens	[69,70]
Vincristine/Vinblastine	Leaves of *Catharanthus roseus* (Madagascar periwinkle)	Breast	Tubulin polymerization inhibition	Approved drug	FDA	New combination regimens ongoing	[71,72]
Topotecan/Irinotecan	Bark of *Camptotheca acuminata* (happy tree)	Investigational in breast	Topoisomerase I inhibition → DNA breaks, apoptosis	Approved for other cancers; off-label in BCD	FDA, EMA	TNBC trials underway	[73,74]
Trastuzuma emtansine (T-DM1)	Maytansinoid (cytotoxin) from *Maytenus ovatus*	HER2+ metastatic breast cancer	HER2-targeted microtubule disruption	Approved drug	FDA, EMA	ADC combination trials	[75,76,77]
Ginsenoside Rg3	Roots/leaves of *Panax ginseng*	Triple-negative breast cancer	Inhibits angiogenesis, glycolysis; reverses resistance	Approved in China; Still under clinical trials (TNBC)	China NMPA	Phase II/III Rg3 + capecitabine	[78,79]
SRg3 + RRg3 Epimers	Derived from *Panax* spp.	Metastatic breast cancer	Stemness, EMT, VEGFR2, AQP 1 inhibition	Phase I/II clinical trials	AACR/SABCS	Studies in metastatic settings	[80,81,82]
Curcumin	Rhizome of *Curcuma longa*	Breast	Inhibits NF κB, STAT3, PI3K/Akt; induces apoptosis	Phase II adjunct trials	NIH, NCI	Oral bioavailability/efficacy trials	[83,84,85,86]
EGCG (Polyphenon E)	Leaves of *Camellia sinensis*	Breast	Epigenetic, PI3K/Akt, anti angiogenic	Not yet approved; Still under investigation for Phase I/II clinical trials	NCI	NCT02580279 ongoing	[7,24,71]
Resveratrol	Skin of grapes (*Vitis vinifera*)	Breast	Activates p53; ROS-induced apoptosis	Not yet approved; Still under investigation for Phase I early-phase trials	NIH, NCI	Early breast cancer trials	[7,87,88,89,90]
Parthenolide	Leaves of *Tanacetum parthenium*	Triple Negative Breast Cancer	NF κB inhibition, ROS generation	Not yet approved; Still under investigation for Preclinical only	NIH, NCI	Microenvironment model exploration	[18,91,92]
Dihydroartemisinin (DHA)	Derived from *Artemisia annua*	Breast	Induces ROS-mediated apoptosis & ferroptosis	Not yet approved; Still under investigation for Preclinical only	NIH, NCI	Combination therapy preclinical	[93,94,95]

**Table 3:** Integration of natural compounds synergizing with conventional therapy.

Natural Compounds	Source Plant/Origin	Type of Cancer	Conventional Therapy Used	Mechanism of Action	Stage of Cancer	Treatment Investigated	Ongoing Research	Study Limitations	Future Directions	References
Curcumin	*Curcuma longa* (turmeric)	HR+, TNBC	Doxorubicin, Paclitaxel, Cisplatin	NF-κB and PI3K/Akt inhibition; ↑ROS; p53-mediated apoptosis; ABC transporter inhibition (MDR reversal)	All stages	In vitro, in vivo; early-phase clinical formulations	Yes	Poor solubility, low bioavailability	Nanoformulations, targeted delivery; controlled trials	[83,84,85,86]
Epigallocatechin gallate (EGCG)	*Camellia sinensis* (green tea)	HR+, TNBC	Doxorubicin, Paclitaxel, Radiotherapy	↑Bax/↓Bcl-2/↑caspase-3; ↑ROS; impaired DNA repair/checkpoints (radiosensitization)	Early– advanced	In vitro, xenografts; limited clinical/epidemiologic	Yes	Low oral bioavailability; dose-dependent effects	Optimized delivery systems; dose-finding trials	[114,117]
Resveratrol	Grapes, berries, peanuts	HR+	Tamoxifen; Radiotherapy	Modulates ER–PI3K/Akt/MAPK crosstalk; restores antiestrogen sensitivity; radiosensitization	Early–locally advanced	In vitro, murine models	Yes	Variable bioavailability; dose/timing sensitivity	Biomarker-guided endocrine combinations; formulation advances	[87,154,174]
Genistein	Soybeans, legumes	HR+	Tamoxifen; Aromatase inhibitors	CDK inhibition; ↑p21; ER/growth factor pathway modulation; delays endocrine resistance	Early– advanced	In vitro, murine models; limited clinical nutrition signals	Yes	Phytoestrogenic antagonism risk	Patient stratification; dosing optimization trials	[163,164,165]
Quercetin	Onion, apple, fruits/vegetables	TNBC, MDR	Doxorubicin, Docetaxel	Inhibits ABC transporters; ↑drug accumulation; enhances caspase-dependent apoptosis	Advanced, resistant	In vitro; limited in vivo	Yes	Sparse clinical data; stability/PK	Resistance-focused preclinical→early clinical studies	[121,123,124,151]
Berberine	*Berberis vulgaris*	TNBC	Cisplatin	G0/G1 arrest; ↑DNA damage response; caspase-3 activation; pro-apoptotic signaling	All stages	In vitro, animals	Yes	Dose-related toxicity	Liposomal/targeted delivery; safety profiling	[160,161,162]
Caffeic acid	Coffee, fruits, vegetables	HR+, TNBC	Paclitaxel	↑ROS; mitochondrial depolarization; G1/S arrest; anti-angiogenic activity	Early– advanced	In vitro, xenografts	Yes	Limited human data	Translational combination trials	[166,167]
Ferulic acid	Whole grains, rice bran	TNBC	Paclitaxel	Suppresses PI3K/Akt/NF-κB; ↓MMP-9; inhibits EMT, invasion, angiogenesis	Advanced/metastatic	In vitro, orthotopic models	Yes	Limited human data	Metastasis-focused translational studies	[175,176,177]
Silymarin	Milk thistle	Mixed subtypes (incl. TNBC)	Paclitaxel (chemosensitization)	NF-κB/MAPK modulation; anti-inflammatory; apoptosis	All stages	In vitro, animals	Yes	Limited human evidence; extract variability	Early-phase clinical trials; standardization	[178]
Thymoquinone	*Nigella sativa*	TNBC	Doxorubicin (chemosensitization)	↑ROS; p38 MAPK activation; mitochondrial apoptosis	Advanced	In vitro, xenografts	Yes	Toxicity concerns at higher doses	Safer analogs; formulation advances	[134,179]
Ginsenoside Rg3	*Panax ginseng*	HR+, TNBC	Cyclophosphamide; multi-drug regimens	Anti-proliferative; immune modulation; anti-angiogenic	All stages	Preclinical; selected clinical contexts regionally	Yes	Cost/supply; standardization	Synthetic production; co-delivery systems	[78,79,81]
Apigenin	Parsley, chamomile	HER2+driven signaling (preclinical)	HER2+pathway targeting contexts	Promotes apoptosis; inhibits STAT3/NF-κB; HER2/neu degradation	Advanced	In vitro	Yes	Poor solubility	Formulation improvements; mechanistic validation	[180,181]
Lycopene	Tomato	HR+	Radiation therapy (adjunct nutrition)	Antioxidant; radioprotective; supports DNA damage response/redox balance	Early-stage	Preclinical; mixed clinical nutrition studies	Yes	Variable absorption; dietary confounders	Dietary guidance; adjunct clinical trials	[182,183]

“↑” and “↓” refer to an increase and decrease in molecule expression respectively; HR+ refers to Hormone Receptors positive; TNBC refers to Triple Negative Breast Cancer; HER2+ refers to Human Epidermal Growth Factor Receptor 2.

## Data Availability

No new data were created or analyzed in this study. Data sharing is not applicable to this article.
